# Necrotic neurons enhance microglial neurotoxicity through induction of glutaminase by a MyD88-dependent pathway

**DOI:** 10.1186/1742-2094-5-43

**Published:** 2008-10-09

**Authors:** Teresa F Pais, Catarina Figueiredo, Rui Peixoto, Maria H Braz, Sukalyan Chatterjee

**Affiliations:** 1 Instituto Gulbenkian de Ciência, Rua da Quinta Grande, 6, 2780-156 Oeiras, Portugal

## Abstract

**Background:**

Microglia are macrophage-like cells that constantly sense the microenvironment within the central nervous system (CNS). In the event of neuronal stress or injury, microglial cells rapidly react and change their phenotype. This response may lead to a deleterious type of microglial activation, which is often associated with neuroinflammation and neurotoxicity in several neuropathological conditions. We investigated the molecular mechanisms underlying triggering of microglial activation by necrotic neuronal damage.

**Methods:**

Primary cultures of microglia were used to study the effect of necrotic neurons on microglial inflammatory responses and toxicity towards cerebellar granule neurons (CGN). The mouse hippocampal cell line, HT22, was used in this study as the main source of necrotic neurons to stimulate microglia. To identify the signal transduction pathways activated in microglia, primary microglial cultures were obtained from mice deficient in Toll-like receptor (TLR) -2, -4, or in the TLR adapter protein MyD88.

**Results:**

Necrotic neurons, but not other necrotic cell types, induced microglial activation which was characterized by up-regulation of: i) MHC class II; ii) co-stimulatory molecules, i.e. CD40 and CD24; iii) β2 integrin CD11b; iii) pro-inflammatory cytokines, i.e. interleukin 6 (IL-6), IL-12p40 and tumor-necrosis factor (TNF); iv) pro-inflammatory enzymes such as nitric oxide synthase (iNOS, type II NOS), indoleamine 2,3-dioxygenase (IDO) and cyclooxygenase-2 (COX-2) and increased microglial motility. Moreover, microglia-conditioned medium (MCM) obtained from cultures of activated microglia showed increased neurotoxicity mediated through the *N*-methyl-D-aspartate receptor (NMDAR). The activation of microglia by necrotic neurons was shown to be dependent on the TLR-associated adapter molecule myeloid differentiation primary response gene (*MyD88*). Furthermore, MyD88 mediated enhanced neurotoxicity by activated microglia through up-regulation of the expression and activity of glutaminase, an enzyme that produces glutamate, which is an NMDAR agonist.

**Conclusion:**

These results show that necrotic neurons activate in microglia a MyD88-dependent pathway responsible for a pro-inflammatory response that also leads to increased neurotoxic activity through induction of glutaminase. This finding contributes to better understanding the mechanisms causing increased neuroinflammation and microglial neurotoxicity in a neurodegenerative environment.

## Background

Necrotic-like neuronal death is associated with the development of several pathologic conditions, including brain ischemia subsequent to stroke or trauma [1], infectious-driven neuroinflammation, e.g. meningitis [2], HIV-associated dementia [3] and cerebral malaria [4], as well as noninfectious-driven neuroinflammation, e.g. multiple sclerosis (MS) [5]. In such pathological conditions microglia, the resident CNS macrophage equivalents, may contribute to neurotoxicity [6]. Upon neuronal injury, microglial cells are activated, acquiring increased motility and phagocytic activity. Moreover, expression of molecules that regulate immune responses, including MHC class I and II, FcγR, and the co-stimulatory molecules B7.1 and B7.2 are also increased during the process of microglial activation. This can be beneficial by removing cellular debris and can also support neuronal survival through the secretion of neurotrophic factors such as bFGF and NGF (reviewed in [7]). On the other hand, this may also lead to unfettered microglial activation with deleterious consequences [8] such as increased production of the pro-inflammatory cytokines IL-1β, TNF, IL-6 and IL-12 [9], neurotoxins including the pro-oxidant molecules nitrogen oxide (NO) and reactive oxygen species (ROS) [10], and metabolites such as quinolinic acid and glutamate [11].

Specific factors released by stressed or damaged neurons, including matrix metalloproteinase-3, (MMP-3), α-synuclein, neuromelanin [8], and ATP [12] have been shown *in vitro *to induce the production of inflammatory factors by microglia. On the other hand, during the last decade several other putative endogenous "danger signals" or "alarmins" [13], released by necrotic but not by apoptotic cells have also been shown to stimulate pro-inflammatory responses in dendritic cells and in monocytes/macrophages. These "danger signals" include the heat shock proteins (hsp) hsp60, hsp70, hsp90, and gp96 [14]; the calcium-binding S100 proteins [15], DNA, proteases, uric acid and the chromosomal protein high-mobility group B1 (HMGB1) (reviewed in [16]). These molecules, referred to as "damage-associated molecular patterns" [13], can be recognized by a series of pattern recognition receptors (PRR) that include TLRs [17], the receptor for advanced glycosylation end products (RAGE) [18] and scavenger receptors [19], but also by other cell surface proteins such as CD40 [20] and CD91 [21], and the intracellular NOD-like receptors (NLRs) family as well, namely NALP-3 [22].

Phenotypic alterations and migration of microglial cells following neuronal cell damage have been extensively reported. However, the molecular mechanisms mediating this activation and microglial cell neurotoxicity require further elucidation. Here we address the effects of neuronal necrosis on microglial activation and neurotoxicity. In this paper we report that necrotic neurons, but not other necrotic cell types, can induce in microglia a pro-inflammatory phenotype that in turns causes NMDAR-mediated toxicity of neurons. This activation of and neurotoxic activity by microglia resulting from stimulation by necrotic neurons is shown to be dependent on MyD88, which mediates the up-regulation of glutaminase.

## Methods

### Mice

C57BL/6, B10scN (which lack the genomic region containing the *tlr-4 *gene), TLR-2 (C57BL/6) and MyD88 (C57BL/6) deficient mice were bred and maintained under specific pathogen-free conditions in our animal facilities in accordance with guidelines from the Animal User and Institutional Ethical Comities of the Instituto Gulbenkian de Ciência.

### Primary cell cultures

#### Microglia

Neonatal microglia cultures were prepared from newborn mice as described previously [23]. Briefly, after removal of the meninges, brains were mechanically disrupted. Cells were cultured in high-glucose Dulbeco's Modified Eagle Medium (DMEM) medium containing Glutamax (Invitrogen, Carlsbad, CA) and supplemented with 10% FBS (Endotoxin less than 10 EU/ml, Invitrogen), 5 μg/ml insulin (Sigma-Aldrich Química, S.A, Portugal), 2.0 mg/ml L-glucose (Sigma) and 1% Penicillin-Streptomycin 50 mM (Invitrogen) for 14–21 days (naïve microglia). Granulocyte-macrophage colony-stimulating factor (GM-CSF) (Peprotech, Rocky Hill, NJ) (GM-CSF-microglia) was added at the beginning of the culture and subsequently every 3 days at a final concentration of 0.25 ng/ml. At day 14, confluent mixed glial cell cultures were shaken for 3 h at 250 rpm. Microglial cells were obtained from the supernatant after filtering through 100-μm cell strainers (BD, Becton, Dickinson and Company, Franklin Lakes, NJ). Cells were resuspended in RPMI 1640 with Glutamax (Invitrogen) supplemented with 5% FBS, 50 μM β-mercaptoethanol and 1% Penicillin-Streptomycin (RMPI-FBS). Cells were plated in 6 well plates (1 × 10^6^) or 24 well plates (2 × 10^5^) for 1 hour. Adherent cells were washed twice with warm Hanks balanced salt solution (HBSS) to remove cell debris and astrocytes. The purity of the primary cultures assessed by CD11b staining and FACS analysis was >90%.

#### Astrocytes and cerebellar granule neurons

An enriched population of primary astrocytes was obtained from the adherent cell population after shaking the mixed cell brain cultures. Primary cerebellar granule neurons (CGN) were prepared from 6-day-old mice as described previously [24]. Briefly, cerebella were dissected and the cells were dissociated and plated at a density of 1 × 10^5 ^neurons/cm^2 ^on poly-L-lysine (0.5 mg/ml, Sigma) coated plates. Cells were maintained in Basal Medium (BME) supplemented with 10% (v/v) FBS, 25 mM KCl (Sigma), 1% penicillin-streptomycin, 1% glutamax and 6 mg/ml glucose. After 24 hr, the medium was replaced to remove dead cells by the above culture medium supplemented with 1% (v/v) N-2 supplement and 50 μM cytosine arabinoside (Sigma) to prevent proliferation of non neuronal cells. Cultures were kept in an incubator at 37°C in 5% CO_2 _and were used after 8 days.

### Induction of cell death

The mouse hippocampal cell line HT22 (kindly provided by J. Pocock) and WHEI 164 cells were cultured in DMEM containing 10% FBS. EL-4 cells were cultured in complete RPMI medium containing 10% FBS. All cell lines tested negative for *Mycoplasma *by PCR. Cells were trypsinized with Trypsin/EDTA (Invitrogen) and washed twice in endotoxin-free PBS. Cells were resuspended in PBS (10^7 ^cells/ml) and a necrotic-like cell death type was induced by 3 cycles of freeze and thaw or mechanically disrupted using a syringe and needle (0.5 × 16 mm). Cells were immediately added to microglial cells or kept on ice until they were used to minimize protease activity. Loss of cell viability was confirmed using trypan-blue (Sigma) (>95% cell death). To induce apoptosis, cells were UV-irradiated in a UV Stratalinker 2400 (Stratagene, La Jolla, CA) for 1 h. The percentage of apoptotic cells was assayed by FACS analysis of annexin staining according to manufactures instructions (BD Biosciences, Pharmingen™).

### Cell stimulation

Primary cultures and cell lines were stimulated with necrotic cells (1:1 or 50–100 μg/ml), IFN-γ (Peprotech) (10–100 U/ml), LPS (Sigma) (100 ng/ml) or Pam3CSK4 (InvivoGen, San Diego, CA) (300 ng/ml) for 24 hours in RPMI 1640 with Glutamax (Invitrogen) supplemented with 5% FBS, 50 μM β-mercaptoethanol and 1% Penicillin-Streptomycin (RMPI-FBS).

### Migration Assay

Cell migration in studies of chemotaxis was assayed as described previously [25]. Briefly, 500 μl of medium containing ATP (100 μM), necrotic cells (2 × 10^5 ^or 50 μg/ml), were applied to 24-well culture plates. Microglial cells (2 × 10^5^) were plated in a 10 mm diameter tissue culture insert with an 8 μm pore size polycarbonate membrane (Nalgene Nunc, Roskilde, Denmark) placed into each well. Cells were incubated for 3 hours at 37°C in 5% CO_2 _atmosphere and migrated cells were collected from the lower chamber. After centrifugation cells were stained with trypan-blue and counted using a hemocytometer (Neubauer chamber) or mixed with 10 μm latex beads (Bechman Coulter, Fullerton, CA) and counted by flow cytometry (BD, FACSCalibur™)

### Flow cytometry

Antibodies against CD11b (M1/70), CD11c (HL3), H-2Kb (AF-88.5), CD86 (GL1), CD40 (3/23), CD80 (16-10A1), CD14 (rmC5-3), CD45 (30-F11) and CD24 (M1/69) were purchased from BD Biosciences (BD, Pharmingen™). The antibody against FcγR (2.4G2) was prepared in house from hybridoma culture supernatants. Microglial cells were detached with cold PBS containing 0.5 mM EDTA and incubated with anti-Fc receptor in FACS buffer (PBS containing 2% FCS and 0.01% NaN_3_) for 30 min at 4°C before staining. After washing cells were incubated with streptavidinallophycocyanin or phycoerythrin (BD, Pharmingen™) for 20 min at 4°C to detect biotinylated antibodies. Cells were then washed and acquired using FACSCalibur™ and CELLQuest™ software (BD Becton, Dickinson and Company). Post acquisition analysis was done with CELLQuest™ or FlowJo™ (Tree Star, Ashland, OR) software.

### Neurotoxicity assay

CGN seeded in glass coverslips were incubated with MCM from non-stimulated cultures or from microglia activated with necrotic neurons at a final concentration of 20%. Where indicated, the MCM was obtained from microglial cells cultured in glutamine-free medium or in medium containing 20 μM of iNOS inhibitor, L-N6-(1-Iminoethyl) lysine (L-Nil, Tocris, UK); 1 mM of IDO inhibitor (Sigma), 1-Mehyl-DL-tryptophan (1-MT, Sigma), or 1,5 mM of glutaminase inhibitor, 6-diazo-5-oxo-L-norleucine (DON, Sigma). NMDA receptor antagonist, 10 μM MK-801 (Tocris), was added to neurons at same time as MCM. To quantify and assess neuronal cell survival, cells were incubated with 10 μg/ml 4'-6'-Diamidino-2-phenylindole (DAPI, Sigma) for 10 min at 37°C and 4 μg/ml propidium iodide (PI, Sigma) for 2 min also at 37°C. PI positive cells (~500) were counted in more than five fields per coverslip and cell death was expressed as the percentage (%) of PI positive cells from the total cells.

### Analysis of cytokines, nitrites (NO_2_^-^) and glutamate

Cytokines were quantified in the supernatant of microglia cultured for 24 hours by ELISA according to manufacturer's instructions: TNF, IL-1β, IL-6 (R&D Systems, Minneapolis, MN) and IL-12 (p40 subunit) (BD OptEIA™). The production of NO by iNOS was measured indirectly by assaying nitrites in the culture supernatant using the Griess reaction. Supernatants were mixed with an equal volume of Griess reagent [26]. Optical density measurements were averaged and converted to micromoles of nitrites per well using a standard curve of sodium nitrite. L-Glutamic acid concentration in the supernatants was determined using the Amplex Red reagent-based assay (Invitrogen) accordingly to the manufacturer's instructions.

### Reverse transcription-PCR Analysis

Microglial cells were treated with IFN-γ (10 U/ml), necrotic HT22 cells (1:1) or left untreated for 24 hours. Total RNA was isolated from microglial cells using Trizol (Invitrogen, USA). RNA was reverse-transcribed from 2 μg of total RNA by 200 U of M-MuLV reverse transcriptase (Fermentas, UK) used accordingly to manufacturer's instructions. PCRs were performed with diluted cDNA using primers for iNOS (sense: 5'-AAG CTG CAT GTG ACA TCG ACC CGT-3'; antisense: 5'-GCA TCT GGT AGC CAG CGT ACC GG-3'), COX-2 (sense: 5'-CAG CAC TTC ACC CAT CAG TT-3'; antisense: 5'-CTG GTC AAT GGA GGC CTT TG-3'), IDO (sense: 5'-GTA CAT CAC CAT GCT GTA TG-3'; antisense: 5'-GCT TTC GTC AAG TCT TCA TTG-3'), Glutaminase (sense: 5'-GTA ATG ATC GTC AAC GGG GGA GGA C-3'; antisense: 5'-CCA GCA AGC CTT GCA ACC TTA ACC TTA ACC A-3') and HPRT (sense: 5'-GTA ATG ATC GTC AAC GGG GGA GGA C-3'; antisense: 5'-CCA GCA AGC TTG CAA CCT TA A CCT TAA CCA-3'). PCR products were resolved on 1% ethidium bromide-stained agarose gel.

### Statistical analysis

Data is expressed as mean ± SD and was analyzed for significance using Student's *t *test. P-values are as follows: *** *p *< 0.005, ** *p *< 0.01 and * *p *< 0.05.

## Results

### Activation of microglia by necrotic neurons

**Figure 1:**
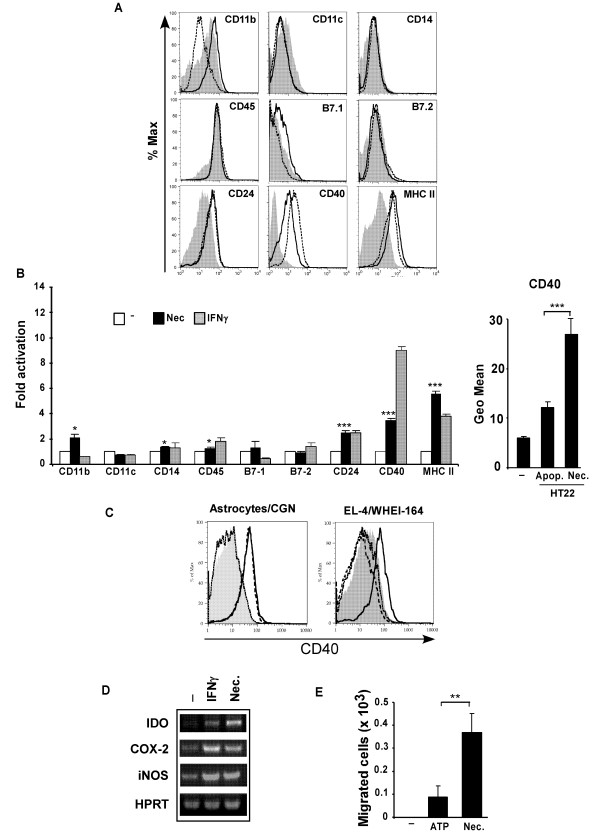
**Necrotic neurons induce expression of activation markers by microglia**. (A) Microglia were stimulated with necrotic HT22 neurons at a ratio of 1:1 for 24 hours and the expression of several activation markers analysed by FACS. The staining intensity is represented on histogram overlays for the mentioned proteins in non-stimulated microglia (grey filled), and in microglia stimulated either with IFN-γ (100 U/ml) (dashed line) or with necrotic neurons (solid line). (B) These results are shown as fold increases ("fold activation") of the Geo Mean when divided by the average expression of triplicates of untreated microglia. The fold increase in the expression of the different markers was compared to the effect on B7.1 expression. Necrotic neurons were significantly more efficient than apoptotic HT22 cells in inducing CD40 expression by microglia (graph on the right). (C) Histogram overlays for CD40 staining intensity of microglia cultured in medium only (grey filled), stimulated with necrotic HT22 neurons (solid line), or incubated with different necrotic cell types: histogram plot on the left, primary cerebellar granule neurons (CGN) (dashed line), astrocyte-enriched cell population (dotted line); histogram plot on the right, EL-4 (dotted line) and WHEI-164 (dashed line) cell lines. (D) Microglial cells were stimulated for 24 hours with necrotic neurons or with IFN-γ (10 U/ml), and the expression of COX-2, IDO and iNOS was analysed by RT-PCR. (E) Microglial cells were plated in an insert containing a membrane with a cut-off of 8 μm. The number of microglial cells migrated through the membrane was quantified 3 hours after adding necrotic HT22 neurons (50 μg/ml) to the lower chamber. ATP (100 μM) was used as a positive control for chemotaxis. Data are representative of at least two experiments done in triplicate. *** p < 0.001; **p < 0.01 and * p < 0.05.

Neuronal cell injury has been previously shown to be associated with up-regulation of several molecules involved in immunological responses of microglia [7]. We investigated the modulation of several cell activation markers [7] by microglial cells responding to necrotic neuronal HT22 cells, a mouse hippocampal cell line [27], using FACS analysis (Figure 1A). Microglial cells responded to necrotic HT22 cells with a 2–6 fold increase in the expression of CD40 and of MHC class II (Figure 1A). The alpha chain of the α_M_β_2 _integrin CD11b and the heat-stable antigen CD24 were also increased, albeit to lesser degrees (~2 fold, p < 0.05 and p < 0.001 respectively) (Figure 1B). Other molecules such as the co-stimulatory molecules B7.1 and B7.2 and CD11c were not up-regulated whereas CD45 and CD14 were poorly induced by necrotic cells (Figure 1B). Apoptotic HT22 neuronal cells induced a lower increase in CD40 expression in microglial cells when compared to necrotic HT22 cells (Figure 1B, right graph). However, some degree of stimulation was detectable with apoptotic HT22 cells, which is probably explained by the high percentage of secondary necrosis which we observed during incubation with microglia (40% after 24 hours) (*data not shown*). Primary cerebellar granule neurons (CGN), but not astrocytes, mimicked the effect of necrotic HT22 cells by inducing CD40 expression (Figure 1C, left panel) and TNF secretion by microglia (71.5 ± 17.6 pg/ml compared to non-detectable values in non-stimulated microglia or microglia incubated with astrocytes). In addition, necrotic cells obtained from mouse EL-4 (lymphoma) or WHEI 164 (fibrosarcoma) cell lines were unable to trigger CD40 expression (Figure 1C, right panel) or TNF (non-detectable values) in microglia. We further evaluated the pro-inflammatory phenotype of microglia upon stimulation with necrotic neurons. Necrotic neurons induced 11- and 9-fold increases in secretion of IL-12p40 and IL-6, respectively (Table 1). Production of TNF and of NO, estimated by the nitrite concentration in the supernatant of microglial cell cultures, were significantly increased by necrotic neurons although ~10-fold lower when compared with the levels induced by other stimuli such as IFN-γ or LPS (data not shown). IL-1β was not induced in microglia by necrotic neurons (Table 1). Furthermore, the mRNA expression levels of enzymes induced during neuroinflammatory conditions triggered by infection or brain injury, such as iNOS [28], IDO [29] and COX-2 [30], were increased in microglia by necrotic neurons (Figure 1D). Finally, necrotic neurons, when compared to a chemotactic stimulus such as ATP [31], induced significantly greater migration of microglial cells, (p < 0.01) seeded on a membrane insert, towards a lower chamber containing necrotic HT22 cells (Figure 1E). In summary, a clear pro-inflammatory phenotype is induced in microglia upon stimulation with necrotic neurons, as is increased microglial motility.

**Table 1:** Parameters of activation in microglia stimulated with necrotic HT22 neurons.

	**Medium**	**Necrotic HT22**
**Cytokine secretion **(pg/ml)		

**IL-1β**	36.6 ± 16.4	37.5 ± 10.3
**TNF**	ND	15.2 ± 5.4*
**IL-12p40**	67.7 ± 5.8	745.7 ± 15.5***
**IL-6**	188.3 ± 4.3	1735.8 ± 85.2***

**NO_2_^- ^(μM)**	ND	12.5 ± 1.6***

Microglial cells were left untreated or stimulated with necrotic HT22. IL-1β, TNFα, IL-12p40, IL-6 and NO_2_^- ^were assayed in the cell supernatant 24 hours after stimulation. ND, non-detectable. *** p < 0.001 and * p < 0.05.

### Microglial activation by necrotic neurons is MyD88-dependent

**Figure 2:**
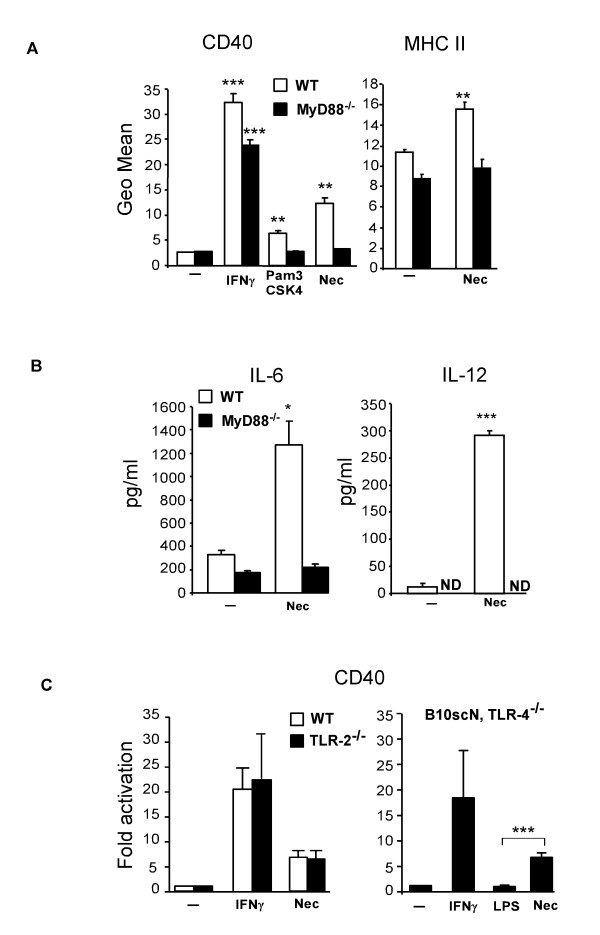
**Microglial response to necrotic neurons is MyD88-dependent**. (A) Microglial cells derived from MyD88-deficient mice were stimulated with necrotic HT22 cells for 24 hours and the expression of CD40 and MHC class II was assessed and compared to that of wild type cells. Pam3CSK4 is a TLR-2 ligand; ND, non-detectable. (B) The levels of IL-6 and IL-12 were quantified in the supernatants of microglial cell cultures by ELISA. (C) Induction of CD40 expression in TLR-2 or TLR-4 deficient microglia stimulated with necrotic neurons. LPS is a specific ligand for TLR-4. Results are shown as mean ± SD of three independent experiments or are representative of at least two experiments. *** p < 0.001; **p < 0.01 and * p < 0.05.

There is mounting evidence suggesting that "danger signals" released from necrotic cells can be recognized by PRR expressed on monocytes/macrophages and dendritic cells. These PRR include, but are not restricted to, members of the TLR family. MyD88 is an adapter protein required for signal transduction by TLRs, with the exception of TLR-3 [32]. We isolated microglial cells from MyD88-deficient mice to assess whether this pathway is involved in signal transduction pathways via which necrotic neurons activate microglial cells. Ablation of MyD88 in microglia cells suppressed microglial activation by necrotic neurons, as assessed by the expression of CD40 or MHC class II (Figure 2A). MyD88-deficient microglia could be activated by IFNγ but not by a specific ligand for TLR-2, Pam3CSK4. Increased IL-6 or IL-12 secretion was also abolished in MyD88-deficient microglia stimulated with necrotic neurons (Figure 2B). These results demonstrate that necrotic neurons are most likely recognized by PRRs expressed on microglia that signal via MyD88 to induce expression of pro-inflammatory genes associated with microglial activation.

TLR-2 and TLR-4 have been identified in macrophages and dendritic cells as putative receptors for endogenous signals such as heat shock proteins hsp70 and gp96 [17] and the chromatin binding HMGB1 protein [33]. We evaluated the putative role of these two TLRs in the microglial response to necrotic neurons using TLR-2^-/- ^mice and B10ScN mice, which lack the genomic region containing the *tlr-4 *gene (Figure 2C). Microglia derived from these mouse lines maintained increased CD40 expression when incubated with necrotic neurons (Figure 2C). These data suggest that necrotic neurons contain ligands for My88-dependent TLR signaling other than TLR-2 or TLR-4 or, alternatively, suggest that several TLRs are activated and interfering with TLR-2 or TLR-4 signaling alone does not impair the microglial response.

### Necrotic neurons enhance microglial glutamate-dependent neurotoxicity

**Figure 3:**
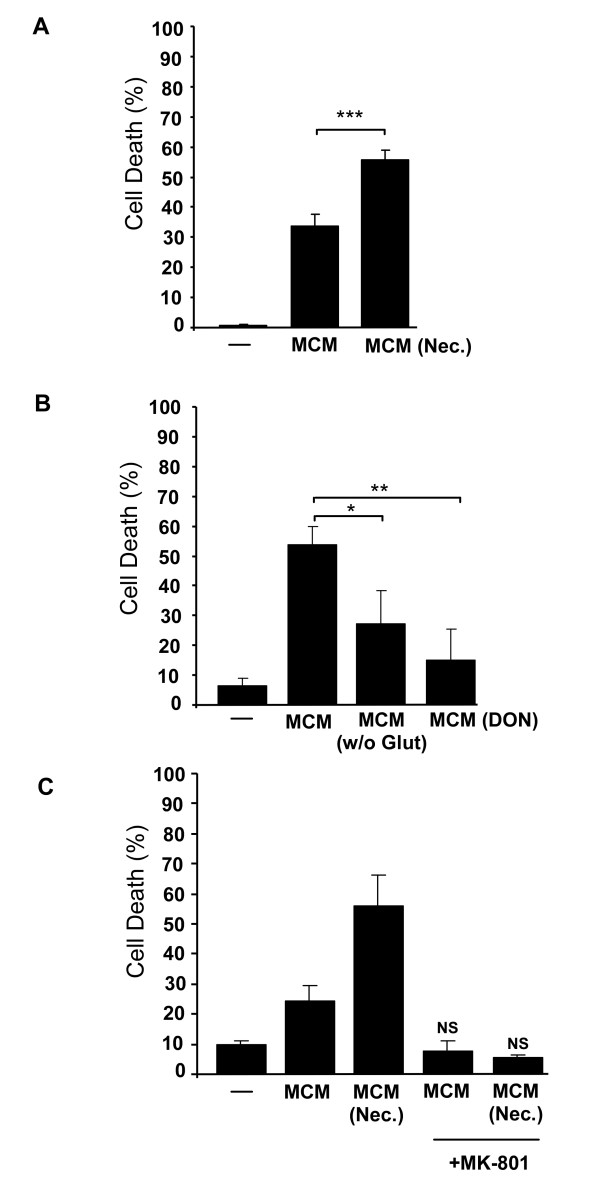
**Necrotic neurons enhance microglial-induced neurotoxicity mediated by NMDAR**. (A) Neurotoxicity of MCM was assayed in cell cultures of CGN after 24 hours. MCM obtained from non-stimulated cells or after stimulation with necrotic neurons for 20 hours, MCM (Nec.), were added to a final concentration of 20% to neuronal cultures. Neuronal viability was analysed by PI incorporation after 24 hours. (B) Neuronal cell death was quantified after exposure to MCM of non-stimulated cells, MCM of microglial cells cultured in glutamine-free medium, MCM (- Glutamine), or cultured with a glutaminase inhibitor, MCM (+DON). (C) The NMDAR inhibitor, MK-801, was added to neuronal cultures at the same time as MCM and neuronal viability was assayed as described before. Results are shown as mean ± SD and are representative of at least two experiments. NS, non-significant. *** p < 0.001 and * p < 0.05.

Microglial cells, depending on their activation phenotype, secrete a variety of factors that include both neurotrophic factors such as bFGF and NGF and neurotoxic metabolites like NO and glutamate [11]. We assessed the neurotoxicity of microglia-conditioned medium (MCM), added at final concentration of 20%, obtained from untreated microglia or from microglia activated with necrotic neurons. Although MCM from untreated microglia exhibited some neurotoxicity, probably due to basal activation levels of this cell type [34], neuronal cell death was significantly increased by 21.5% (p < 0.005) when neurons were incubated with MCM from activated microglia (MCM (Nec.), Figure 3A). The neurotoxicity of MCM was due to the microglial conditioning since exposure of neurons to fresh medium at the same ratio resulted in a lesser, and non-significant, increase in cell death (13.4 ± 10.5% of neurons compared to 8.3 ± 3.0% in the absence of medium). We next investigated the basis for MCM-induced neurotoxicity. This was neither abolished when MCM was heat inactivated before exposing it to neurons, nor by inhibition of the microglial enzymes iNOS and IDO, which are responsible for the formation of two neurotoxic agents, NO and QA, respectively (data not shown). On the other hand, MCM obtained from microglial cells cultured in glutamine-free medium (- Glutamine) or in the presence of the glutaminase inhibitor, DON (+ DON), showed significantly reduced neurotoxicity (Figure 3B). These results suggested that cell death could be mediated by glutamate through NMDAR. To test this hypothesis, MCM from non-stimulated or stimulated microglia were added together with MK-801, an antagonist of NMDAR. In both conditions, the antagonist completely abolished the MCM-induced neurotoxicity, clearly showing that the mechanism of neurotoxicity mediated by microglia is NMDAR-dependent. Altogether these results show that necrotic neurons cause an over-activation of microglia with consequent NMDAR-mediated neurotoxicity, most likely mediated through increased production of glutamate.

### Deletion of MyD88 in microglia blocks up-regulation of glutaminase and neurotoxicity by necrotic neurons

**Figure 4:**
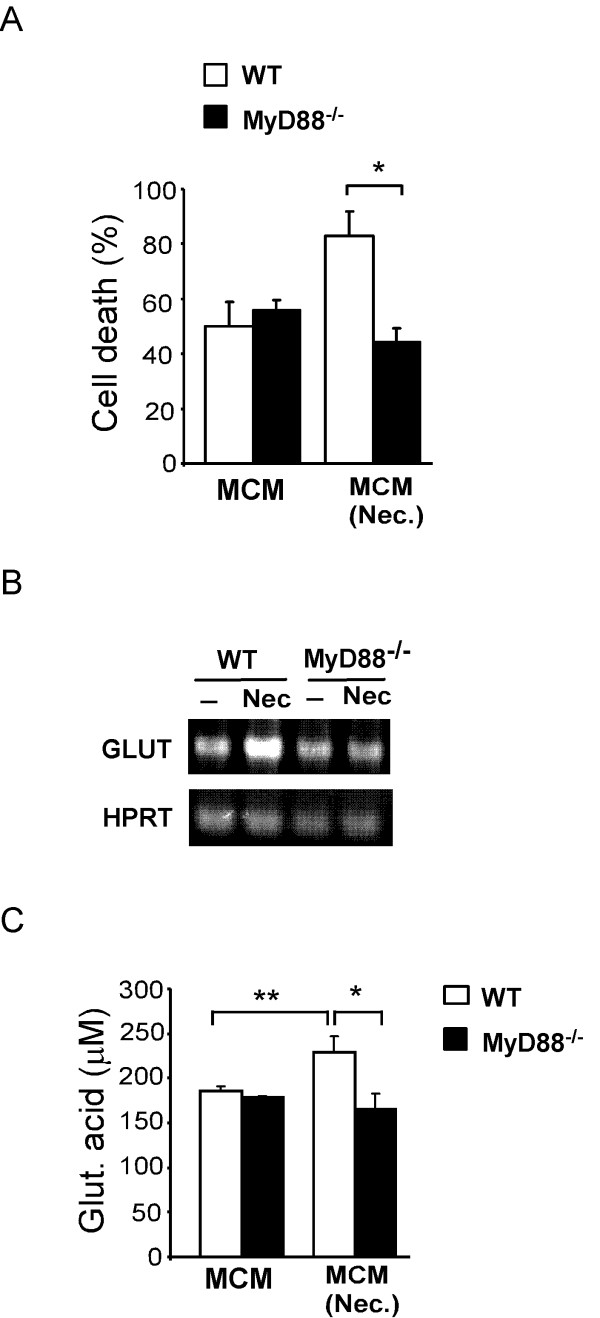
**MyD88-dependent induction of neurotoxicity and glutaminase expression in microglia**. (A) Neurotoxicity of MCM from wild type (WT) and MyD88-deficient microglia cultures either non-stimulated (MCM) or treated with necrotic HT22 neurons (MCM (Nec.)). (B) RT-PCR analysis of glutaminase expression of wild type or MyD88-deficient microglia treated or not treated with necrotic HT22 neurons. (C) Analysis of L-Glutamic acid levels in MCM of microglial cell cultures. Results are shown as mean ± SD and are representative of at least two experiments. *** p < 0.001 and * p < 0.05.

As we have demonstrated above, necrotic neurons activate microglia through a MyD88-dependent pathway. Consequently, we investigated whether the enhanced neurotoxicity of stimulated microglia was affected by the absence of MyD88. In contrast to wild type microglia, the increase in neurotoxicity associated with activation of microglia by necrotic neurons was completely abrogated in the absence of MyD88 expression in microglia (Fig 4A). When primary cerebellar neurons were cultured with 20% MCM from microglia pre-stimulated with necrotic neurons there was a 33% increase in cell death compared to cerebellar neurons cultured with MCM from non-stimulated cultures. In contrast, MCM from MyD88-deficient microglia treated with necrotic neurons did not show any increase in neurotoxicity. Since we have shown (Figure 3) that neurotoxicity is dependent on glutaminase activity, we analysed the mRNA expression levels of glutaminase and glutamate in supernatants of microglia from both wild type and MyD88-deficient microglia. We observed that the observed increase in neurotoxicity correlated with up-regulation of glutaminase mRNA expression in wild type microglia activated by necrotic neurons (Figure 4B). This increase was not observed in MyD88-deficient microglia, and this explains the inability of these microglia to produce enhanced neurotoxicity upon stimulation with necrotic neurons. Concomitantly, levels of glutamate were increased in MCM of wild type, but not MyD88-deficient, microglia stimulated with necrotic neurons 4C.

Taken together our results demonstrate that neurotoxicity induced by necrotic neurons in microglia is mediated through MyD88-dependent signaling cascades, leading to increased glutaminase expression and consequent increase in glutamate-mediated neurotoxicity.

## Discussion

Microglia and perivascular macrophages/dendritic cells in the CNS become activated upon neuronal injury. The goal of the present study was to understand the pathways of microglial activation induced by neuronal necrosis, and the effects of such activation on microglia-mediated neurotoxicity. We demonstrate that necrotic neurons induce several markers of microglial activation such as co-stimulatory molecules, MHC class II, pro-inflammatory cytokines and enzymes and enhanced motility. This activation, which involves a MyD88-dependent signalling pathway, also increases NMDAR-dependent microglial neurotoxicity by up-regulation of glutaminase expression and activity.

Bacteria-derived products are known to induce neuronal cell death through the activation of the MyD88 pathway in microglia [35]. Here we demonstrate that "molecular danger patterns" exposed by necrotic neurons also activate microglia and enhance microglial neurotoxicity by a MyD88-dependent pathway. However, in the absence of TLR-2, TLR-4 and TLR-9 (data not shown) which signal through MyD88, the response of microglia to necrotic neurons is not affected. Either necrotic neurons activate other than those tested, or more than a single TLR are redundantly activated in response to the necrotic stimuli. In the later case, only by blocking a common signaling point, as in MyD88-deficient microglia, would activation of microglia by necrotic neurons be highly compromised as we have observed using assays of CD40 and MHC II expression and IL-6 and IL-12 production (Figure 3). A mechanism involving activation of NLRs by ligands exposed by necrotic neurons cannot be excluded since these PRRs induce IL-1β and IL-18 which act through MyD88. [36]. However, we did not detect an increase in IL-1β secretion by microglia activated with necrotic neurons.

Microglia activated with necrotic neurons clearly show a pro-inflammatory phenotype dependent on MyD88. This type of response by microglia may contribute to enhanced neuropathogenesis after necrotic neuronal cell death. Interestingly, it has been shown recently that MyD88-deficient mice, but not TLR4/TLR2-deficient mice, show reduced brain inflammatory responses and decreased lesions after cold-induced cortical injury [37]. However, pro-inflammatory mediators may have diverse effects after neuronal injury caused by brain ischemia. Whereas inhibition of COX-2 is neuroprotective [38], the role of pro-inflammatory cytokines such IL-6 and TNF is ambiguous in this context, with both deleterious and beneficial effects having been reported [39].

We have demonstrated that glutaminase expression is regulated by a MyD88-dependent pathway. By bioinformatics analysis using PROMO software [40,41], we have identified three putative DNA-binding sites for the transcription factor Activation Protein-1 (AP-1) at 20 bp, 240 bp and 300 bp upstream of the start codon. Since a MyD88-dependent pathway was shown to activate AP-1, which together with NF-kB controls the induction of pro-inflammatory cytokines [42], AP-1 may be one of the putative transcription factors involved in the induction of glutaminase expression. 

It has been reported that HSP60, present in the supernatant of total necrotic CNS cells, activates TLR4 on microglia leading to increased microglial neurotoxicity towards cortical neurons. This effect could be partially but significantly reduced by inhibiting iNOS activity, suggesting that other neurotoxic mechanisms may be involved [43]. In our study, iNOS was induced in microglia activated by necrotic neurons but NO did not play a significant role in mediating the killing of CGN. This probably reflects different susceptibilities to neurotoxic molecules on the part of different neuronal cell populations. Of the variety of neurotoxic molecules that activated microglia can secrete [11], we clearly show that glutamate is the sole candidate as the agent that enhances neurotoxicity of CGN through NMDAR in microglia activated with necrotic neurons. These results are in accordance with previously described mechanisms of macrophage/microglial neurotoxicity [44-46]. Moreover, it has been shown that excessive activation of this receptor contributes to a large number of neurological disorders associated with microglial activation [47,48].

## Conclusion

We demonstrate that necrotic neurons induce a pro-inflammatory and glutaminase-mediated neurotoxic response by microglia. Both mechanisms are dependent on the adapter signalling protein MyD88 and may be highly relevant to increased neuropathology in the context of brain injury. Therefore, the MyD88-dependent pathway and glutaminase activity may constitute potential targets for new pharmacological strategies to control massive neuroinflammation and neurodegeneration.

## Competing interests

The authors declare that they have no competing interests.

## Authors' contributions

SC is the Principal Investigator and Head of the laboratory and the group, and conceptualized and initiated the project. TFP is a post doctoral fellow who orchestrated and carried out the work with the help of the following graduate students, and wrote the paper. CF carried out the neurotoxicity assays. RP participated in the FACS analysis of microglial activation and was involved in revising the manuscript. MHB carried out the migration assays. All authors read and approved the final manuscript.
